# Decrease in naturally occurring antibodies against epitopes of Alzheimer’s disease (AD) risk gene products is associated with cognitive decline in AD

**DOI:** 10.1186/s12974-023-02750-9

**Published:** 2023-03-15

**Authors:** Dongmei Gu, Luchun Wang, Nan Zhang, Huali Wang, Xin Yu

**Affiliations:** 1 Clinical Research Division, Dementia Care and Research Center, Peking University Institute of Mental Health (Sixth Hospital), Beijing, China; 2 Beijing Dementia Key Lab, NHC Key Laboratory of Mental Health, National Clinical Research Center for Mental Disorders (Peking University), Beijing, China; 3 Department of Neurology, Tianjin Medical University General Hospital, Tianjin, China

**Keywords:** Alzheimer’s disease, Plasma biomarkers, Natural antibodies, Phagocytosis, Immunity

## Abstract

**Background:**

Naturally occurring antibodies (NAbs) are germline-encoded immunoglobulins that can bind to and clear out self-neo-epitopes as well as apoptotic and necrotic cells. However, NAbs pathological relevance in Alzheimer’s disease (AD) is not well-understood.

**Methods:**

Twenty-eight candidate proteins encoded by AD-associated genes were selected for this study based on a number of selection criteria, including preferential expression in the brain and B-lymphocyte cells. The levels of NAbs in plasma were analyzed according to their epitopes in age- and gender-matched cognitively normal subjects (CN, *n* = 56), subjects with mild cognitive impairment (MCI, *n* = 16) and subjects with AD (*n* = 56). We aimed to study the levels of their NAbs in plasma and their associations with cognitive decline in individuals with AD.

**Results:**

Of the 28 antigens tested, 17 showed decreased NAbs in individuals with AD; in particular, NAb-TREM2 had an area under the ROC curve of 0.806, with the highest sensitivity (0.370) at 95% specificity among all 28 tests. Further protein–protein interaction networks and functional enrichment analysis suggested that target genes were enriched in AD-related pathological processes classified under “Alzheimer’s disease”, “neurodegenerative disease” and “amyloidosis”. The “Alzheimer’s disease” and “neurodegenerative disease” clusters, which converged on the initial “recognition” step of microglial phagocytosis, showed the best diagnostic performance for AD.

**Conclusions:**

This study suggests a decline in the function of the adaptive immune system in AD, and the levels of circulating NAbs are likely to serve as biomarkers for surveilling the progression of AD.

**Supplementary Information:**

The online version contains supplementary material available at 10.1186/s12974-023-02750-9.

## Background

Alzheimer’s disease (AD) is the most common cause of dementia in elderly people and is becoming increasingly prevalent worldwide [1]. Traditionally, the most important pathomechanism of AD is hypothesized to be the amyloid-β (Aβ) cascade, in which the brain harbors abnormal accumulation of Aβ cleaved from amyloid precursor protein (APP) into toxic extracellular plaques; another pathomechanism is the intracellular accumulation of neurofibrillary tangles made of hyperphosphorylated and misfolded tau protein [2]. The breakdown of brain proteostasis plays a central role in AD pathogenesis, including neurodegeneration and neuroinflammation triggered by glial cells [3]. Recent studies have suggested the involvement of innate and adaptive immune systems in the development of AD, raising the neuroinflammation hypothesis [4]. Genome-wide association studies (GWAS) in a late-onset Alzheimer’s disease (LOAD) cohort and a combination of AD cohorts confirmed numerous genetic factors that govern diverse cellular and molecular pathways involved in immune responses; in addition, studies of the human leukocyte antigen (HLA) region confirmed the neurological and immune-mediated disease haplotype HLA-DR15 as a risk factor for LOAD [5, 6]. Most of the related risk genes are expressed in at least one type of brain cell and in peripheral immune cells [7]. RNA-seq results suggest that AD risk genes identified by GWAS are downregulated in several tissues in the AD-affected brain, including the cortex, cerebellum, hippocampus, basal ganglia, and amygdala. However, these genes are “overexpressed” in adolescent and adult brains [8]. There is a more aggressively skewed distribution of peripheral immune cells in AD patients than in healthy aging individuals, with features such as a reduction in B-cell receptor repertoire diversity and switched memory B cells, accompanied by a diminished antibody response to antigen challenge [9, 10].

Naturally occurring antibodies (NAbs) stands for physiological antibodies, or autoantibodies generated in healthy humans, in contrast to those induced by exogenous antigens [11]. NAbs are produced spontaneously by B-lymphocytes without antigenic exposure from infection or vaccination; these antibodies carry out diverse functions in maintaining immune homeostasis and in the regulation of autoimmune responses [12]. Immunological tests in the plasma and cerebrospinal fluid (CSF) of AD patients identified significantly decreased NAbs against Aβ-related epitopes [13]. In addition, NAbs against self-antigens related to neurotransmitters, microglia, lipids, and vessels are also considered autoimmune factors associated with AD [12, 13]. This is consistent with the biological function modules suggested by GWAS and RNA-seq of human brains [5, 6]. Some pilot studies have identified NAbs that can either accelerate or prevent neurodegeneration, depending on the target antigens and cell types involved [12]. Although the profiles and pathophysiological roles of NAbs in the pathogenesis of AD are far from clear, it makes sense to clarify the level of NAbs associated with high-risk factors in AD.

A recent large-scale GWAS highlighted the presence of diverse mechanisms in AD pathogenesis and suggested candidate targets for diagnostic and therapeutic development [5, 6]. In this study, we integrated GWAS results with eQTLs (expression quantitative trait loci) for AD and limited them to a certain set of target risk genes in AD-associated pathways. We further aimed to profile the levels of these NAb-IgGs against linear epitopes of those AD risk genes and assess their pathological relevance in AD cohorts. By focusing on linear epitopes rather than whole proteins, we improved our chances of identifying specific AD-related diagnostic targets.

## Methods

### Participants

Fifty-six patients with AD, 16 patients with mild cognitive impairment (MCI) and 56 age- and gender-matched cognitively normal (CN) subjects were recruited from the Dementia Care and Research Center at Peking University Institute of Mental Health and memory clinic at Tianjin Medical University General Hospital. This study was approved by the Ethics Committee of Peking University Sixth Hospital (Institute of Mental Health) and Tianjin Medical University General Hospital. Written informed consent was obtained from all participants.

The inclusion criteria for the AD group were as follows: (1) met the criteria of the National Institute on Aging and Alzheimer’s Association (NIA-AA) for probable AD [14]; (2) had more than 6 years of education; (3) ranged from 50 to 85 years of age; (4) had a Clinical Dementia Rating (CDR) score = 0.5–2, and Mini-Mental State Examination (MMSE) score less than 27; (5) had a score of ≤ 4 on the modified Hachinski ischemic scale.

The inclusion criteria for the MCI group were as follows: (1) met the NIA-AA criteria [14] for MCI due to AD; (2) had more than 6 years of education; (3) ranged from 50 to 85 years of age; and (4) had a CDR score of 0.5, with no cognitive or functional impairment that was severe enough to meet the criteria for dementia.

The inclusion criteria for the cognitively normal control group were as follows: (1) aged between 50 and 85 years; (2) had more than 6 years of education; and (3) did not have memory or cognitive complaints or objective cognitive impairment. Exclusion criteria included (1) having neurological or mental disorders potentially affecting cognitive function, such as depression, schizophrenia, or an alcohol-related disorder; (2) major medical problems, such as tumors, cerebrovascular events; and (3) systematic or neurological autoimmune diseases.

Both AD and MCI patients were confirmed by an amyloid-positive result on a ^11^C-labeled Pittsburgh Compound-B (PiB) PET scan. Cognitive function was assessed with the MMSE for all participants.

### Plasma preparation

Three milliliters of venous blood was drawn into a 5 mL tube of saline with ethylenediaminetetraacetic acid (EDTA). The separation of plasma was performed within 3 h of sample collection. The whole blood was centrifuged at 2500×*g* for 15 min at 4 °C. The plasma was transferred to storage tubes (Solarbio, Science & Technology Co., Ltd. BJ) as 500 µL aliquots and stored at − 80 °C until assayed.

### Detection of antibodies against linear peptide antigens

Target proteins encoded by the genes harboring or next to the index SNPs associated with AD confirmed by two previous large-scale fine-mapping analyses in AD using GWAS and eQTL data [5, 6]. In brief, a total of 43 AD-associated genes with genome-wide significance were selected in this study, and their locations in the human genome, target genes, and physiological functions were summarized in Additional file 1: Table S1. Target proteins encoded by candidate genes were identified from the NCBI protein database (http://www.ncbi.nlm.nih.gov/protein). Linear peptide antigens were designed based on the in silico prediction of HLA-II epitopes in the Immune Epitope Database (http://www.iedb.org/). A total of 28 out of 43 proteins had detectable linear epitopes, and their sequence information is listed in Additional file 1: Table S2. An in-house enzyme-linked immunosorbent assay (ELISA) was optimized for plasma NAbs detection [15]. The linear peptide antigen was synthesized by a solid-phase chemical method with a purity of > 95% and dissolved in 67% acetic acid to obtain a 5 mg/mL stock solution (stored at − 20 °C). The working solution was diluted just before use with coating buffer (0.1 M phosphate buffer containing 0.15 M NaCl and 1 mM EDTA) to 10 µg/mL. Corning 96-Well Microtiter EIA Plates (CLS2510-50EA/CLS2509-50EA, Sigma-Aldrich) were coated in 0.1 mL/well of the antigen and incubated for 90 min at 37 °C. The plates were washed three times using 200 μL of wash buffer A (0.1 M phosphate buffer containing 0.15 M NaCl) and blocked using 0.2 mL (10 μg/mL) cysteine/well in coating buffer for 1 h at room temperature. Plates were washed twice with 200 μL of wash buffer A and dried at 40 °C for 2 h. Plates, once dried, were sealed with sealing film and stored at 4 °C until use. The plates were restored to room temperature just before use and were washed twice with 200 μL of wash buffer B [phosphate-buffered saline (PBS) containing 0.1% Tween-20] in each well to rehydrate. The plasma sample (including positive control, PC) was diluted 1:100 in assay buffer (PBS containing 0.5% bovine serum albumin), and 50 μL of the sample was loaded into each sample well; 50 μL of assay buffer was added to each negative control (NC) well. Following incubation at 37 °C for 120 min, the plate was washed 3 times with 200 μL of wash buffer B, and 50 μL of peroxidase-conjugated goat anti-human IgG Fc (ab98624, Abcam, Cambridge, UK) diluted 1:40,000 in assay buffer was then added and incubated for 60 min at room temperature. After incubation, the plate was washed 3 times with 200 μL of wash buffer B, 50 μL of 3,3′,5,5′-tetramethylbenzidine (PR1200, Solarbio) was added, and the plate was incubated in the dark for 20 min before 50 μL of the stop solution was added (C1058, Solarbio).

Each antigen was tested separately, and each test panel was evenly distributed with 8 AD subjects, 2–3 MCI subjects, and 8 control subjects. The optical density (OD) of each well was then measured within 10 min with a plate reader at 450 nm with a reference wavelength of 620 nm. All samples were tested in duplicate, and the specific binding ratio (SBR) was calculated for each sample using the following formula:

$${\text{SBR}} = {{\left( {{\text{OD}}_{{{\text{sample}}}} - {\text{OD}}_{{{\text{NC}}}} } \right)} \mathord{\left/ {\vphantom {{\left( {{\text{OD}}_{{{\text{sample}}}} - {\text{OD}}_{{{\text{NC}}}} } \right)} {\left( {{\text{OD}}_{{{\text{PC}}}} - {\text{OD}}_{{{\text{NC}}}} } \right)}}} \right. \kern-0pt} {\left( {{\text{OD}}_{{{\text{PC}}}} - {\text{OD}}_{{{\text{NC}}}} } \right)}}.$$

The coefficient of variation (CV) was used to represent an inter-assay deviation estimated with pooled plasma samples, which was called quality control (QC) sample. The QC samples were randomly collected, mixed and aliquoted from > 100 subjects (control, MCI and AD) and tested on every 96-well plate.

### Statistical analysis

The characteristics of the patients and control subjects were summarized using descriptive statistics. Continuous variables were described as medians, and categorical data were summarized as absolute frequencies and percentages. The categorical variable of gender was analyzed with the chi-square (*χ*^2^) test. Age was analyzed using one-way analysis of variance (ANOVA). Education and MMSE scores were analyzed using the Kruskal–Wallis test. Comparative group *P* values were determined via one-way ANOVA for each level of NAbs. Post hoc* P* values were determined using Tukey’s adjustment for multiple comparisons. *P* values were compared to a Bonferroni-adjusted threshold based on the number of tests per hypothesis: one for each biomarker tested against a clinical diagnosis [*α* = 0.05/number of tests (28), 0.002]. Receiver operating characteristic (ROC) analyses were used to determine diagnostic accuracy. AD or MCI as the outcome was the dependent variable, and levels of NAbs or their combination was the variable in the logistic regression. SPSS version 20.0 (IBM) and GraphPad Prism 9 software were used for statistical analysis.

## Results

### Demographics of all participants

There were no significant differences in age or gender among the three groups (*P* > 0.05). The median duration of education and MMSE score were significantly lower in patients with AD and MCI than in CNs (*P* < 0.05). The demographic and clinical characteristics of all participants were summarized in Table 1.

**Table 1:** Demographic and clinical characteristics of the study participants

Variable	CN (*n* = 56)	MCI (*n* = 16)	AD (*n* = 56)	*χ*^2^/*F*	*P* values
Female, no. (%)	36 (64.3)	11 (68.8)	42 (75.0)	1.52	0.467
Age, mean ± SD, y	69.1 ± 7.3	71.8 ± 9.2	71.6 ± 7.9	1.70	0.186
Education, median (IQR), y	15 (12.0–15.0)	12 (8.0–16.0)	9 (9.0–14.0)	17.34	< 0.001
MMSE, median (IQR)	29 (29.0–30.0)	24 (21.0–27.0)	19 (14.0–23.0)	86.320	< 0.001

Chi-square (*χ*^2^) test was used for analysis of gender. Age was analyzed using one-way analysis of variance (ANOVA). Education and MMSE score were analyzed using Kruskal–Wallis test

*IQR* interquartile range, *AD* Alzheimer’s disease, *CN* cognitively normal controls, *MMSE* Mini-Mental State Examination, *SD* standard deviation

### Levels of plasma NAbs in the AD, MCI and CN groups

**Fig. 1:**
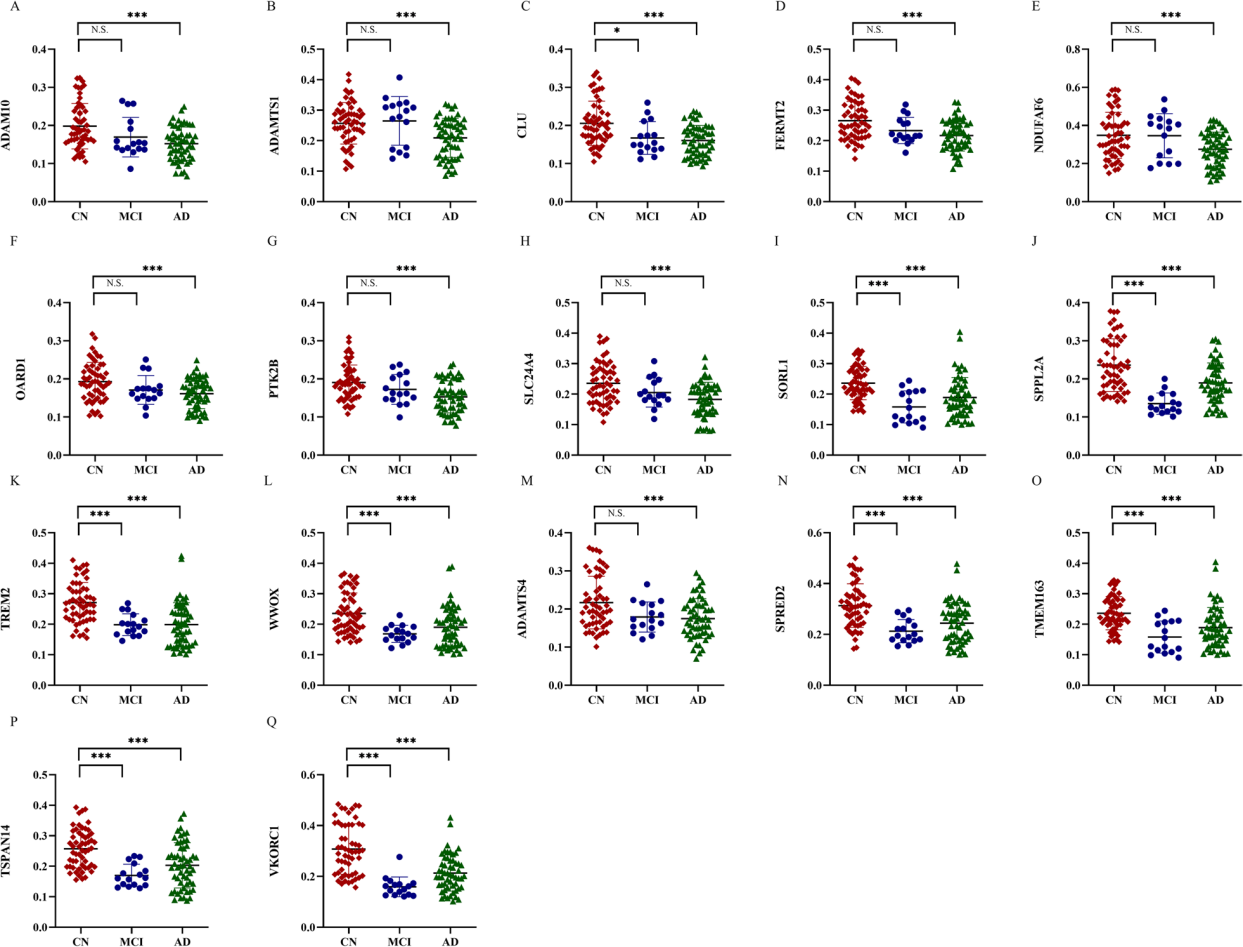
Plasma levels of NAbs in the CN, MCI and AD groups. Plasma levels of NAbs in each group were analyzed using one-way analysis of variance (ANOVA). N.S. denotes no statistical difference. *Denotes nominal significance only at *P* < 0.05. ***Denotes significance after Bonferroni correction at *P* < 0.002 [*α* = 0.05/number of tests (28), 0.002]. *NAbs* natural antibodies, *AD* Alzheimer’s disease, *MCI* mild cognitive impairment, *CN* cognitively normal controls

**Fig. 2:**
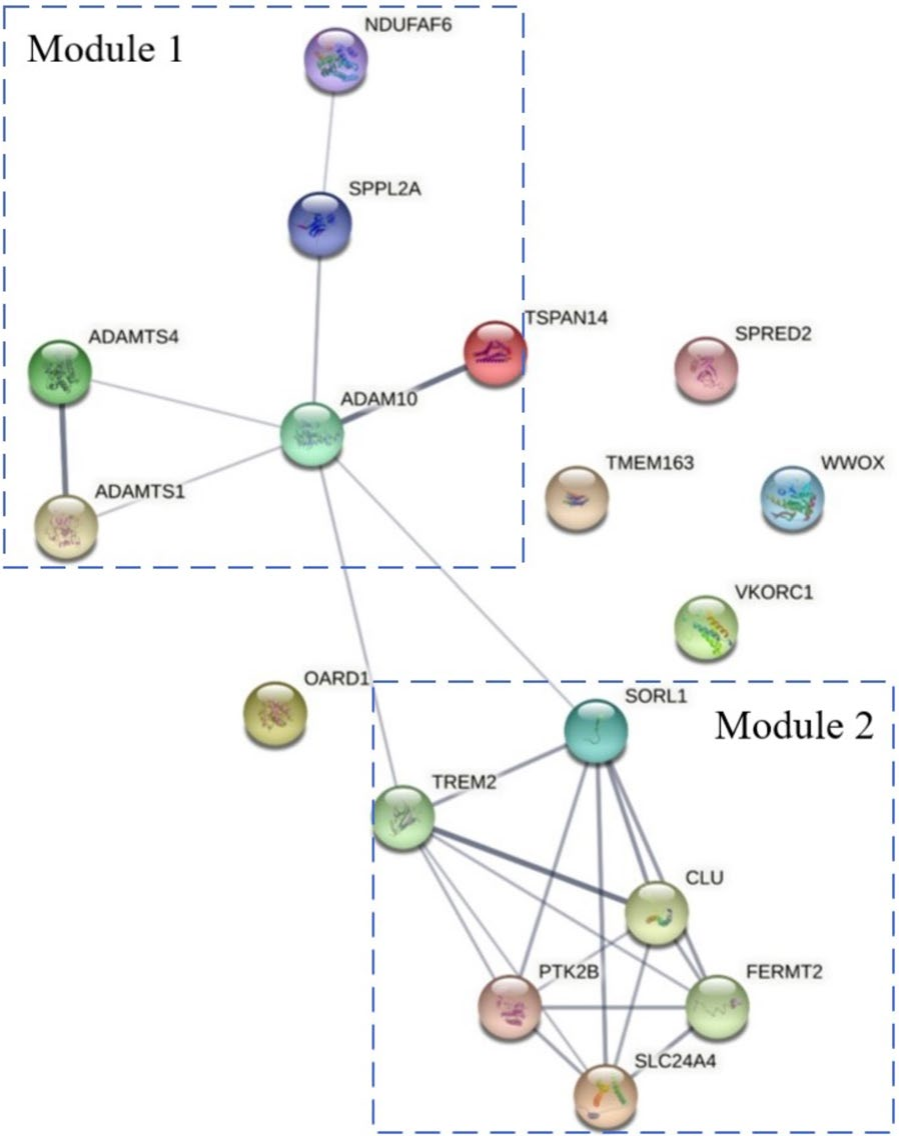
Protein–protein interaction networks and functional enrichment analysis of 17 differential targets. There are two major protein–protein interaction network modules, both of which are significantly associated with AD-related biological processes and pathological processes. The module 1 includes six differential targets (ADAM10, ADAMTS1, ADAMTS4, NDUFAF6, SPPL2A, TSPAN14), while six differential targets (CLU, FERMT2, PTK2B, SLC24A4, SORL1, TREM2) in module 2. In biological process, the module 1 genes are enriched in “integrin-mediated signaling pathway”; module 2 genes are enriched in processes including “glial cell proliferation”, “regulation of neurofibrillary tangle assembly” and “integrin-mediated signaling pathway”. Both module genes are enriched in AD-related pathological processes including “Alzheimer’s disease”, “Neurodegenerative disease” and “Amyloidosis”

The distribution of inter-assay CVs for all antigens ranged from 5 to 14%, which met the general requirement of ELISA batch variation, i.e., less than 20% (Additional file 1: Table S3). Levels of plasma NAbs in the AD, MCI and CN groups were shown in Table 2 and Fig. 2. The levels of all 28 NAbs were lower in the AD group than in the CN group (*P* < 0.05). The levels of 17 NAbs (ADAM10, ADAMTS1, CLU, FERMT2, NDUFAF6, OARD1, PTK2B, SLC24A4, SORL1, SPPL2A, TREM2, WWOX, ADAMTS4, SPRED2, TMEM163, TSPAN14 and VKORC1), named the “Total 17 NAbs” panel, were still significantly lower in the AD group than in the CN group after Bonferroni correction (*P* < 0.002) (Fig. 1). The levels of 10 NAbs (CLU, SORL1, SPPL2A, TREM2, WWOX, APH1B, SPRED2, TMEM163, TSPAN14 and VKORC1) were lower in the MCI group than in the CN group (*P* < 0.05). The levels of 8 NAbs (SORL1, SPPL2A, TREM2, WWOX, SPRED2, TMEM163, TSPAN14 and VKORC1) were still significantly lower in the MCI group than in the CN group after Bonferroni correction (*P* < 0.002).

**Table 2:** Levels of NAbs in the CN, MCI and AD groups and correlation analysis between plasma levels of NAbs and MMSE scores in three groups

	NAbs	Comparison groups								Correlation analysis	
		CN (*n* = 56)	MCI (*n* = 16)	AD (*n* = 56)	*F*	*P* value	CN vs. MCI *P* value	CN vs. AD *P* value	MCI vs. AD *P* value	*r*	*P* value
1	ADAM10	0.20 ± 0.060	0.17 ± 0.052	0.15 ± 0.044	10.977	4.100E−05	0.130	2.300E−05	0.476	0.391	2.000E−04
2	ADAMTS1	0.26 ± 0.068	0.26 ± 0.080	0.21 ± 0.064	8.432	3.670E−04	0.912	0.001	0.013	0.277	0.002
3	BIN1	0.29 ± 0.090	0.32 ± 0.106	0.25 ± 0.083	5.183	0.007	0.517	0.038	0.018	0.219	0.015
4	CASS4	0.28 ± 0.079	0.30 ± 0.091	0.24 ± 0.076	4.959	0.008	0.757	0.024	0.038	0.215	0.016
5	CD2AP	0.22 ± 0.062	0.21 ± 0.057	0.19 ± 0.058	2.932	0.057	0.795	0.045	0.601	0.294	0.001
6	CLU	0.21 ± 0.058	0.17 ± 0.043	0.16 ± 0.040	12.568	1.100E−05	0.018	1.000E−05	0.885	0.361	3.800E−05
7	FERMT2	0.27 ± 0.063	0.23 ± 0.043	0.22 ± 0.051	10.917	4.300E−05	0.183	2.500E−05	0.552	0.297	0.001
8	INPP5D	0.25 ± 0.077	0.26 ± 0.096	0.21 ± 0.070	4.721	0.011	0.833	0.024	0.053	0.168	0.063
9	IQCK	0.27 ± 0.085	0.27 ± 0.099	0.23 ± 0.074	4.462	0.013	0.994	0.014	0.175	0.182	0.043
10	MEF2C	0.30 ± 0.082	0.32 ± 0.106	0.25 ± 0.079	6.296	0.002	0.763	0.008	0.020	0.186	0.039
11	NDUFAF6	0.35 ± 0.121	0.35 ± 0.116	0.28 ± 0.090	7.145	0.001	0.998	0.001	0.056	0.201	0.025
12	OARD1	0.19 ± 0.050	0.17 ± 0.038	0.16 ± 0.039	7.231	0.001	0.196	0.001	0.717	0.284	0.001
13	PICALM	0.19 ± 0.051	0.18 ± 0.045	0.16 ± 0.034	6.708	0.002	0.732	0.001	0.222	0.245	0.006
14	PTK2B	0.19 ± 0.046	0.17 ± 0.039	0.15 ± 0.042	10.440	6.400E−05	0.312	3.400E−05	0.257	0.269	0.003
15	SLC24A4	0.24 ± 0.067	0.21 ± 0.048	0.18 ± 0.056	10.689	5.200E−05	0.183	2.800E−05	0.395	0.284	0.001
16	SORL1	0.24 ± 0.054	0.16 ± 0.052	0.19 ± 0.066	14.667	2.000E−06	2.700E–05	1.510E–04	0.164	0.275	0.002
17	SPPL2A	0.24 ± 0.069	0.14 ± 0.028	0.19 ± 0.052	21.228	1.193E−08	4.337E−08	1.580E−04	0.004	0.341	1.240E−04
18	TREM2	0.27 ± 0.066	0.20 ± 0.036	0.20 ± 0.071	18.808	2.416E−10	1.180E−04	5.523E−09	1.000	0.441	3.800E−07
19	WWOX	0.24 ± 0.067	0.17 ± 0.028	0.19 ± 0.065	10.787	4.800E−05	0.001	0.001	0.432	0.271	0.002
20	ADAMTS4	0.22 ± 0.069	0.18 ± 0.040	0.17 ± 0.052	7.641	0.001	0.066	0.001	0.966	0.245	0.006
21	APH1B	0.21 ± 0.063	0.17 ± 0.035	0.18 ± 0.046	7.299	0.001	0.011	0.004	0.734	0.207	0.021
22	APOE	0.24 ± 0.074	0.20 ± 0.046	0.20 ± 0.058	6.044	0.003	0.105	0.003	0.983	0.223	0.013
23	CCDC6	0.24 ± 0.064	0.21 ± 0.049	0.20 ± 0.051	5.304	0.006	0.215	0.005	0.898	0.227	0.011
24	NCK2	0.26 ± 0.067	0.22 ± 0.044	0.23 ± 0.072	3.899	0.023	0.104	0.039	0.913	0.200	0.026
25	SPRED2	0.31 ± 0.086	0.21 ± 0.046	0.24 ± 0.081	15.349	1.000E−06	5.300E−05	3.200E−05	0.343	0.336	1.350E−04
26	TMEM163	0.27 ± 0.088	0.15 ± 0.036	0.20 ± 0.073	19.892	3.153E−08	7.686E−07	1.300E−05	0.068	0.323	2.510E−04
27	TSPAN14	0.26 ± 0.063	0.17 ± 0.037	0.20 ± 0.074	15.357	1.000E−06	2.200E−05	7.800E−05	0.185	0.372	2.100E−05
28	VKORC1	0.31 ± 0.099	0.16 ± 0.039	0.21 ± 0.071	29.317	3.622E−11	1.368E−08	4.149E−08	0.055	0.392	7.000E−06

Values are provided as mean ± SD. The NAbs levels in plasma of AD patients, MCI patients and CNs were compared with one-way analysis of variance (ANOVA) and Post hoc* P* values were determined using Tukey’s adjustment for multiple comparisons. Correlation analysis between plasma levels of NAbs and MMSE scores was analyzed using Spearman correlation analysis. *P* values were compared against a Bonferroni-adjusted given the number of tests per hypothesis: one for testing biomarkers against a clinical diagnosis [*α* = 0.05/number of tests (28), 0.002]

*NAbs* natural antibodies, *AD* Alzheimer’s disease, *MCI* mild cognitive impairment, *CN* cognitively normal controls, *MMSE* Mini-Mental State Examination

### Association of plasma NAbs levels with cognitive function

To examine whether NAb levels were associated with cognitive function, we evaluated the correlations between NAb levels and MMSE scores among the AD, MCI and CN groups. Levels of 11 NAbs among the “Total 17 NAbs” panel (ADAM10, CLU, FERMT2, OARD1, SLC24A4, SPPL2A, TREM2, SPRED2, TMEM163, TSPAN14, VKORC1) were positively correlated with MMSE score (*r* = 0.284–0.441, *P* < 0.002), as shown in Table 2. The NAb-TREM2 levels showed the strongest correlation with cognitive function (*r* = 0.441,* P* < 0.001).

### Assessment of the diagnostic utility of individual plasma NAbs

We assessed the diagnostic utility of the plasma NAbs above using ROC analyses, as shown in Table 3. Each of the 17 NAbs in the “Total 17 NAbs” panel (ADAM10, ADAMTS1, CLU, FERMT2, NDUFAF6, OARD1, PTK2B, SLC24A4, SORL1, SPPL2A, TREM2, WWOX, ADAMTS4, SPRED2, TMEM163, TSPAN14 and VKORC1) could differentiate AD from age- and gender-matched CN individuals with ROC areas under the curve (AUCs) ranging from 0.662 to 0.806. NAb-TREM2 showed the best diagnostic performance of AD (AUC = 0.806), with the highest sensitivity (against 95% specificity) up to 0.370.

**Table 3:** Assessment of diagnostic utility of NAbs of “total” panels by ROC analysis

NO	NAbs	CN vs. AD			CN vs. MCI		
		AUC (95% CI)	Cutoff	Sensitivity^against 95% specificity^	AUC (95% CI)	Cutoff	Sensitivity^against 95% specificity^
1	ADAM10	0.722 (0.629–0.816)	0.118	0.259	NA	NA	NA
2	ADAMTS1	0.686 (0.587–0.785)	0.128	0.111	NA	NA	NA
3	CLU	0.731 (0.639–0.823)	0.126	0.259	NA	NA	NA
4	FERMT2	0.724 (0.631–0.817)	0.182	0.278	NA	NA	NA
5	NDUFAF6	0.662 (0.562–0.763)	0.185	0.204	NA	NA	NA
6	OARD1	0.694 (0.596–0.791)	0.113	0.185	NA	NA	NA
7	PTK2B	0.730 (0.637–0.824)	0.125	0.333	NA	NA	NA
8	SLC24A4	0.715 (0.62–0.809)	0.145	0.333	NA	NA	NA
9	SORL1	0.760 (0.671–0.849)	0.149	0.296	0.842 (0.734–0.949)	0.148	0.500
10	SPPL2A	0.683 (0.585–0.781)	0.151	0.241	0.938 (0.876–0.999)	0.150	0.750
11	TREM2	0.806 (0.727–0.884)	0.164	0.370	0.834 (0.735–0.934)	0.164	0.188
12	WWOX	0.709 (0.614–0.804)	0.148	0.333	0.814 (0.711–0.916)	0.146	0.188
13	ADAMTS4	0.673 (0.574–0.773)	0.135	0.259	NA	NA	NA
14	SPRED2	0.748 (0.658–0.837)	0.202	0.333	0.856 (0.765–0.947)	0.202	0.563
15	TMEM163	0.751 (0.662–0.84)	0.149	0.333	0.910 (0.836–0.983)	0.149	0.563
16	TSPAN14	0.725 (0.632–0.818)	0.169	0.370	0.884 (0.797–0.970)	0.166	0.563
17	VKORC1	0.796 (0.715–0.877)	0.178	0.352	0.952 (0.895–1.000)	0.178	0.813

*NAbs* natural antibodies, *AD* Alzheimer’s disease, *MCI* mild cognitive impairment, *CN* cognitively normal controls, *NA* not applicable, *ROC* receiver operating characteristic, *AUC* area under the curve

Eight lower NAbs mentioned above (SORL1, SPPL2A, TREM2, WWOX, SPRED2, TMEM163, TSPAN14 and VKORC1) in the MCI group than in the CN group could differentiate MCI from age- and gender-matched CN individuals with ROC AUCs ranging from 0.814 to 0.952. Among them, NAb-VKORC1 showed excellent diagnostic performance for MCI (AUC = 0.952) with the highest sensitivity (against 95% specificity) of up to 0.813.

### Functional modules of the 17 differential targets

To further clarify the potential biological functions of the 17 differential targets, we generated protein–protein interaction networks and conducted functional enrichment analysis using the STRING online database (https://string-db.org/). There are two major protein–protein interaction network modules, both of which are significantly associated with AD-related biological processes and pathological processes (Fig. 2). Module 1 included six differential targets (ADAM10, ADAMTS1, ADAMTS4, NDUFAF6, SPPL2A, TSPAN14), while there were six differential targets (CLU, FERMT2, PTK2B, SLC24A4, SORL1, TREM2) in module 2. In the biological process category, module 1 genes were enriched in the “integrin-mediated signaling pathway”; module 2 genes were enriched in processes including “glial cell proliferation”, “regulation of neurofibrillary tangle assembly” and “integrin-mediated signaling pathway”. Both module genes were enriched in AD-related pathological processes, including “Alzheimer’s disease”, “neurodegenerative disease” and “amyloidosis”. Each protein in the six functional processes was listed in Fig. 2 and Table 4.

**Table 4:** Assessment of diagnostic utility of each panel by ROC analysis

Category	Term description	Matching proteins in network	CN vs. AD		CN vs. MCI	
			AUC (95% CI)	Sensitivity^against 95% specificity^	AUC (95% CI)	Sensitivity^against 95% specificity^
Total	Total 17 NAbs	17 NAbs	0.975 (0.947–1.000)	0.926	1.000 (1.000–1.000)	1.000
GO process	Glial cell proliferation	CLU, TREM2, PTK2B	0.914 (0.862–0.965)	0.685	0.885 (0.798–0.972)	0.500
GO process	Regulation of neurofibrillary tangle assembly	SORL1, CLU	0.821 (0.742–0.900)	0.554	0.872 (0.776–0.968)	0.500
GO process	Integrin-mediated signaling pathway	ADAM10, ADAMTS1, FERMT2, PTK2B	0.782 (0.699–0.865)	0.429	0.675 (0.540–0.810)	0.250
DISEASES	Alzheimer’s disease	SORL1, ADAM10, CLU, TREM2	0.929 (0.884–0.973)	0.722	0.916 (0.843–0.989)	0.688
DISEASES	Neurodegenerative disease	SORL1, ADAM10, CLU, TREM2, WWOX	0.928 (0.883–0.973)	0.759	0.942 (0.888–0.996)	0.750
DISEASES	Amyloidosis	SORL1, ADAM10, CLU	0.838 (0.764–0.912)	0.518	0.877 (0.786–0.968)	0.500

*NAbs* natural antibodies, *AD* Alzheimer’s disease, *MCI* mild cognitive impairment, *CN* cognitively normal controls, *ROC* receiver operating characteristic, *AUC* area under the curve

### Fitted diagnostic power analysis of functional enrichment genes

**Fig. 3:**
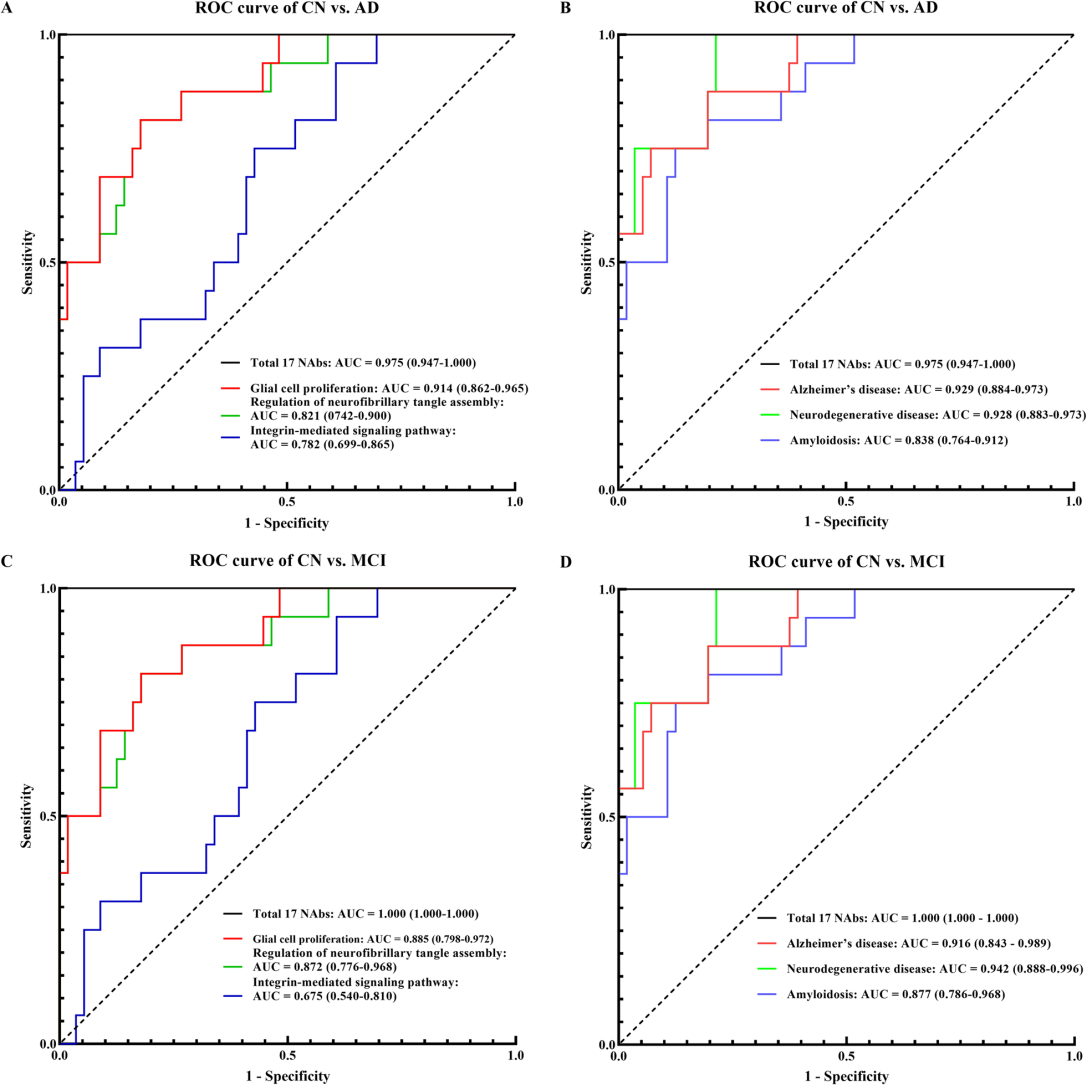
Assessment of diagnostic utility of different panels of NAbs by ROC analysis. Different panels of NAbs could differentiate AD (**A** and** B**) or MCI (**C** and** D**) from age- and gender matched CN individuals with diagnostic utility. *NAbs* natural antibodies, *AD* Alzheimer’s disease, *MCI* mild cognitive impairment, *CN* cognitively normal controls, *ROC* receiver operating characteristics, *AUC* area under the curve

We next assessed the fitted diagnostic powers of NAbs associated with genes enriched in the above six functional processes (Fig. 3). Between the AD and CN groups, the above NAbs involved in biological processes of “glial cell proliferation”, “regulation of neurofibrillary tangle assembly” and “integrin-mediated signaling pathway” could differentiate AD from age- and gender-matched CN individuals with ROC AUCs of 0.914 (0.862–0.965), 0.821 (0742–0.900) and 0.782 (0.699–0.865) (Fig. 3A and Table 4), respectively. The above NAbs involved in AD-related pathological processes, including “Alzheimer’s disease”, “neurodegenerative disease” and “amyloidosis”, could differentiate AD from age- and gender-matched CN individuals with ROC AUCs of 0.929 (0.884–0.973), 0.928 (0.883–0.973) and 0.838 (0.764–0.912) (Fig. 3B and Table 4), respectively. Apart from the “Total 17 NAbs” cluster, the NAb clusters for “Alzheimer’s disease” (SORL1, ADAM10, CLU and TREM2) and “Neurodegenerative disease” (SORL1, ADAM10, CLU and TREM2 and WWOX) showed the best diagnostic performance for AD with sensitivity (against 95% specificity) up to 0.759.

Between the MCI and CN groups, the above NAbs involved in biological processes of “glial cell proliferation”, “regulation of neurofibrillary tangle assembly” and “integrin-mediated signaling pathway” could differentiate MCI from age- and gender-matched CN individuals with ROC AUCs of 0.885 (0.798–0.972), 0.872 (0.776–0.968) and 0.675 (0.540–0.810) (Fig. 3C and Table 4), respectively. The above NAbs, involved in AD-related pathological processes classified under “Alzheimer’s disease”, “neurodegenerative disease” and “amyloidosis”, could differentiate MCI subjects from age- and gender-matched CN individuals with ROC AUCs of 0.916 (0.843–0.989), 0.942 (0.888–0.996) and 0.877 (0.786–0.968), respectively (Fig. 3D and Table 4). As above, the NAb clusters for “Alzheimer’s disease” and “neurodegenerative disease” showed the best diagnostic performance with sensitivity (against 95% specificity) up to 0.750.

## Discussion

In this study, we profiled plasma levels of NAb-IgG against linear epitopes of proteins derived from 28 AD-associated genes. We integrated GWAS results with eQTLs and limited them to those target genes thought to be relevant to AD [5, 6]. Gene enrichment analysis showed that these genes were distributed in established biological pathways involved in AD pathogenesis, including endocytosis, immune responses, phagocytosis, and the complement cascade [5]. Among them, the 17 NAbs that were reduced in AD were involved in some biological processes related to AD pathology, such as glial cell proliferation, regulation of neurofibrillary tangle assembly. In general, the NAb-IgG levels were lower in AD patients, suggesting that there may be a defect in humoral immunity in AD. The decline in NAbs on these pathways implies a loss of protective function and the disruption of immune homeostasis [12]. It is possible that during aging and progression of AD pathogenesis, immune surveillance and homeostasis are out of balance in the central nervous system (CNS) [16, 17]. The NAb-IgG levels were significantly reduced in brain regions severely affected by AD [18]. This reduction in NAb levels may enable Aβ accumulation and failure of Aβ clearance [4, 19], thereby facilitating AD progression. The decline of NAbs targeting AD known key proteins suggests that NAbs may influence biological functions (such as glial cell proliferation and regulation of neurofibrillary tangle assembly) through the regulation of the function of its target gene proteins, which constitutes a significant regulatory dimension in the pathogenesis of AD.

Among the antibodies considered in the present study, NAb-TREM2 was significantly reduced in AD and MCI and showed the best individual diagnostic performance for AD, with an AUC of up to 0.806. NAb-TREM2 also showed the strongest correlation with cognitive function. TREM2 (triggering receptor expressed on myeloid cells 2) is expressed in microglia in the brain [20]. This receptor may play a multifaceted role in maintaining normal microglial functions in the homeostatic AD brain [21]. Anti-human TREM2 antibodies were shown to boost microglial responses to Aβ in vitro [22], moderate Aβ plaque load after short-term treatment [23], promote microglial proliferation and attenuate the neurotoxic effects of Aβ plaques after long-term administration [24]. Therefore, anti-TREM2 antibodies, such as AL002, are thought to be promising candidates for AD therapy [24].

These 17 NAbs were further classified into six clusters according to protein–protein interaction networks and functional enrichment analysis (Fig. 2). Both the “Alzheimer’s disease” and “neurodegenerative disease” clusters showed the best diagnostic performance for AD and MCI. The common risk variants and proteins in those two clusters are SORL1, ADAM10, CLU, and TREM2, which converge on microglia and the endolysosomal network. Microglia are resident macrophages in the CNS and play key roles in brain homeostasis and function [19]. Microglia play a beneficial role in generating anti-Aβ antibodies and stimulating the clearance of amyloid plaques [25]. Microglia can clear Aβ peptides via phagocytosis [19]. This broad biological process includes recognition of released and membrane-tethered chemotactic signals recognized by phagocytic receptors; rearrangements of the actin cytoskeleton leading to formation of a phagocytic cup; digestion of engulfed material in endolysosomal compartments; and finally activation of transcription factors that enhance the clearance transcriptional program [26]. CLU, as a phagocytic substrate, can be coated with Aβ and then recognized by microglial phagocytic receptors including TREM2 and SORL1. ADAM10 is involved in TREM2 shedding [5, 6]. Thus, our study suggests that NAbs of the common risk variant and proteins in both the “Alzheimer’s disease” and “neurodegenerative disease” clusters converged on the initial “recognition” step of phagocytosis on microglia.

In addition, although some novel NAbs target proteins and risk genes (NDUFAF6, OARD1, SLC24A4, SPPL2A, ADAMTS4, SPRED2, TMEM163, TSPAN14, VKORC1, and others) are reported to be associated with AD in GWAS [5, 6], the mechanisms underlying AD pathogenesis are unclear. We first report the alteration of their NAbs levels in AD progression, which can provide a clue for the identification of novel mechanisms underlying AD.

This study has several strengths. First, unlike many blood proteins and lipids, antibodies have remarkably stable concentrations in the blood, which ensures that their production and detection will be largely independent of circadian as well as day-to-day variations in production [27]. Previous studies have shown that individual autoantibody profiles are influenced by age, gender and the presence or absence of disease [28]. In this study, the AD group and the normal control group were matched for age and gender as in other similar studies [29]. Second, the targeting genes were from GWAS, which provided strong support for investigating the mechanisms of AD by applying multiple methods of pathway analysis. Therefore, the autoantibodies screened are more likely to be associated with AD.

There are several limitations in this study. First, the sample size of the MCI group was relatively small than that in AD and control groups. The study sample were retrieved from the existing cohort. Only 16 subjects with MCI who were confirmed with PiB-PET were available during our study period. Further studies with more PiB-PET confirmed MCI subjects are warranted to verify the findings in the present study. In addition, it is impossible to fully control the confounding effects of lifestyle factors, such as smoking, diets, and substance use on the NAbs levels measured. Second, comparing the correlation of NAbs levels in plasma and cerebrospinal fluid (CSF) may provide further insight into the potential biological mechanisms of the NAbs in AD pathophysiology. However, our cohort only used PiB-PET to confirm the diagnosis of Alzheimer’s disease, while did not collect CSF sample. In addition, clarifying the cytological characteristics of NAbs-derived B cell populations may elucidate the potential function of NAbs in AD. However, the present study was based on the blood sample collected in the clinical cohort. The sample collected could not allow us to count cells. Third, researchers may argue that the approach used in the present study might miss the information of NAbs that is against conformational epitopes, because we used linear peptide epitopes against NAbs rather than whole proteins. However, most targets in the present study were first reported in GWAS. Currently, lack of detailed 3D protein crystal structure information of these targets has posed great difficulties in designing the assay panel against corresponding conformational epitopes of the whole proteins. Using recombinant proteins may be considered as an alternative way. However, this method may introduce dozens of linear and conformational epitopes and led to detection of mixed signals of all epitopes. Given the possible cross-reactivity of NAbs, such “mixed signals” may bring more false positive and false negative results. Finally, it will be meaningful to test these NAbs in long-term follow-up cohorts which accompanied by a gradual health or pathological aging processes.

## Conclusion

We discovered 17 NAbs that had reduced plasma concentrations in the AD group and were correlated with cognitive function. Among them, NAb-TREM2 showed the best individual diagnostic performance for AD. The “Alzheimer’s disease” and “neurodegenerative disease” clusters, which converged on the initial “recognition” step of microglial phagocytosis, showed the best diagnostic performance for AD and MCI. Our results suggest that AD-associated NAbs are potential blood-based biomarkers for the diagnosis of AD or MCI and promising candidates for AD therapeutic target. Further studies are warranted to explore the potential biological relevance of these NAbs in AD.

## Supplementary Information

**Additional file 1: Table S1.** Summary of the identified loci reaching genome-wide significance. **Table S2.** Epitope of target proteins encoded by candidate genes. **Table S3.** Analysis of the inter-assay deviation between plates.

## Data Availability

The data sets generated and analyzed during the current study are available from the corresponding author on reasonable request.

## References

[1] Frisoni GB, Altomare D, Thal DR, Ribaldi F, van der Kant R, Ossenkoppele R, Blennow K, Cummings J, van Duijn C, Nilsson PM (2022). The probabilistic model of Alzheimer disease: the amyloid hypothesis revised. Nat Rev Neurosci.

[2] Knopman DS, Amieva H, Petersen RC, Chetelat G, Holtzman DM, Hyman BT, Nixon RA, Jones DT (2021). Alzheimer disease. Nat Rev Dis Prim.

[3] Perluigi M, Barone E (2022). Aberrant protein networks in Alzheimer disease. Nat Rev Neurol.

[4] Li Y, Laws SM, Miles LA, Wiley JS, Huang X, Masters CL, Gu BJ (2021). Genomics of Alzheimer’s disease implicates the innate and adaptive immune systems. Cell Mol Life Sci.

[5] Schwartzentruber J, Cooper S, Liu JZ, Barrio-Hernandez I, Bello E, Kumasaka N, Young AMH, Franklin RJM, Johnson T, Estrada K (2021). Genome-wide meta-analysis, fine-mapping and integrative prioritization implicate new Alzheimer’s disease risk genes. Nat Genet.

[6] Kunkle BW, Grenier-Boley B, Sims R, Bis JC, Damotte V, Naj AC, Boland A, Vronskaya M, van der Lee SJ, Amlie-Wolf A (2019). Genetic meta-analysis of diagnosed Alzheimer’s disease identifies new risk loci and implicates Abeta, tau, immunity and lipid processing. Nat Genet.

[7] Consortium GT, Laboratory DA, Coordinating Center-Analysis Working G, Statistical Methods groups-Analysis Working G, Enhancing Gg, Fund NIHC, Nih/Nci, Nih/Nhgri, Nih/Nimh, Nih/Nida, et al. Genetic effects on gene expression across human tissues. Nature. 2017;550:204–13. 10.1038/nature24277 PMC5776756 29022597

[8] Miller JA, Ding SL, Sunkin SM, Smith KA, Ng L, Szafer A, Ebbert A, Riley ZL, Royall JJ, Aiona K (2014). Transcriptional landscape of the prenatal human brain. Nature.

[9] Naradikian MS, Hao Y, Cancro MP (2016). Age-associated B cells: key mediators of both protective and autoreactive humoral responses. Immunol Rev.

[10] Martinez-Jimenez CP, Eling N, Chen HC, Vallejos CA, Kolodziejczyk AA, Connor F, Stojic L, Rayner TF, Stubbington MJT, Teichmann SA (2017). Aging increases cell-to-cell transcriptional variability upon immune stimulation. Science.

[11] Spath PJ, Lutz HU (2012). Naturally occurring antibodies/autoantibodies in polyclonal immunoglobulin concentrates. Adv Exp Med Biol.

[12] Sim KY, Im KC, Park SG (2020). The functional roles and applications of immunoglobulins in neurodegenerative disease. Int J Mol Sci.

[13] Wu J, Li L (2016). Autoantibodies in Alzheimer’s disease: potential biomarkers, pathogenic roles, and therapeutic implications. J Biomed Res.

[14] Jack CR, Bennett DA, Blennow K, Carrillo MC, Dunn B, Haeberlein SB, Holtzman DM, Jagust W, Jessen F, Karlawish J (2018). NIA-AA research framework: toward a biological definition of Alzheimer’s disease. Alzheimers Dement.

[15] Fritzler MJ, Hudson M, Choi MY, Mahler M, Wang M, Bentow C, Milo J, Baron M, Canadian Scleroderma Research G (2018). Bicaudal D2 is a novel autoantibody target in systemic sclerosis that shares a key epitope with CENP-A but has a distinct clinical phenotype. Autoimmun Rev.

[16] Rustenhoven J, Drieu A, Mamuladze T, de Lima KA, Dykstra T, Wall M, Papadopoulos Z, Kanamori M, Salvador AF, Baker W (2021). Functional characterization of the dural sinuses as a neuroimmune interface. Cell.

[17] Harry GJ (2013). Microglia during development and aging. Pharmacol Ther.

[18] Zhang L, Xu J, Gao J, Chen P, Yin M, Zhao W (2019). Decreased immunoglobulin G in brain regions of elder female APOE4-TR mice accompany with Abeta accumulation. Immun Ageing.

[19] Wang S, Colonna M (2019). Microglia in Alzheimer’s disease: a target for immunotherapy. J Leukoc Biol.

[20] Ulland TK, Colonna M (2018). TREM2—a key player in microglial biology and Alzheimer disease. Nat Rev Neurol.

[21] Qin Q, Teng Z, Liu C, Li Q, Yin Y, Tang Y (2021). TREM2, microglia, and Alzheimer’s disease. Mech Ageing Dev.

[22] Cheng Q, Danao J, Talreja S, Wen P, Yin J, Sun N, Li CM, Chui D, Tran D, Koirala S (2018). TREM2-activating antibodies abrogate the negative pleiotropic effects of the Alzheimer’s disease variant Trem 2(R47H) on murine myeloid cell function. J Biol Chem.

[23] Schlepckow K, Monroe KM, Kleinberger G, Cantuti-Castelvetri L, Parhizkar S, Xia D, Willem M, Werner G, Pettkus N, Brunner B (2020). Enhancing protective microglial activities with a dual function TREM2 antibody to the stalk region. EMBO Mol Med.

[24] Wang S, Mustafa M, Yuede CM, Salazar SV, Kong P, Long H, Ward M, Siddiqui O, Paul R, Gilfillan S (2020). Anti-human TREM2 induces microglia proliferation and reduces pathology in an Alzheimer’s disease model. J Exp Med.

[25] Cai Z, Hussain MD, Yan LJ (2014). Microglia, neuroinflammation, and beta-amyloid protein in Alzheimer’s disease. Int J Neurosci.

[26] Podlesny-Drabiniok A, Marcora E, Goate AM (2020). Microglial phagocytosis: a disease-associated process emerging from Alzheimer’s disease genetics. Trends Neurosci.

[27] DeMarshall CA, Nagele EP, Sarkar A, Acharya NK, Godsey G, Goldwaser EL, Kosciuk M, Thayasivam U, Han M, Belinka B (2016). Detection of Alzheimer’s disease at mild cognitive impairment and disease progression using autoantibodies as blood-based biomarkers. Alzheimers Dement (Amst).

[28] DeMarshall C, Oh E, Kheirkhah R, Sieber F, Zetterberg H, Blennow K, Nagele RG (2019). Detection of early-stage Alzheimer’s pathology using blood-based autoantibody biomarkers in elderly hip fracture repair patients. PLoS ONE.

[29] Shim SM, Koh YH, Kim JH, Jeon JP (2022). A combination of multiple autoantibodies is associated with the risk of Alzheimer’s disease and cognitive impairment. Sci Rep.

