# Supplementary material for: Decrease in naturally occurring antibodies against epitopes of Alzheimer’s disease (AD) risk gene products is associated with cognitive decline in AD
Source: J Neuroinflammation. 2023 Mar 15;20:74. doi: 10.1186/s12974-023-02750-9 (PMC10018846; doi:10.1186/s12974-023-02750-9)
Supplement: Supplementary file 1 — Additional file 1: Table S1. Summary of the identified loci reaching genome-wide significance. Table S2. Epitope of target proteins encoded by candidate genes. Table S3. Analysis of the inter-assay deviation between plates. [file 12974_2023_2750_MOESM1_ESM.docx]

Table S1: Summary of the identified loci reaching genome-wide significance

| **No.** | **PMID** | **Variant** | **OR** | **Chr.** | **Position^a^** | **Major/ minor alleles** | **P value** | **Closest gene^b^** | **Biological function** |
| --- | --- | --- | --- | --- | --- | --- | --- | --- | --- |
| 1 | 33637963 | rs4575098 | 1.04 | 1 | 161155392 | G/A | 4.30E-08 | ADAMTS4 | Metal ion binding; Metalloendopeptidase activity; Metallopeptidase activity; Involved in extracellular matrix disassembly and organization. |
| 2 | 31417202; 33637963 | rs6733839 | 1.11 | 2 | 127892810 | C/T | 2.10E−44; 1.10E-54 | BIN1 | Nucleocytoplasmic adaptor; Involved in synaptic vesicle endocytosis; Interact with dynamin, synaptojanin, endophilin, and clathrin. |
| 3 | 31417202; 33637963 | rs10933431 | 1.05 | 2 | 233981912 | C/G | 3.4E-09; 1.41E-10 | INPP5D | Affecting multiple signaling pathways; involved in nuclear inositol phosphate signaling processes; A negative regulator of myeloid cell proliferation and survival. |
| 4 | 33637963 | rs143080277 | 1.43 | 2 | 106366056 | T/C | 1.28E-12 | NCK2 | A member of the NCK family of adaptor proteins; Bind and recruit various proteins involved in the regulation of receptor protein tyrosine kinases; be involved in cytoskeletal reorganization |
| 5 | 33637963 | rs268134 | 0.96 | 2 | 65608363 | A/G | 1.54E-08 | SPRED2 | Regulate growth factor-induced activation of the MAP kinase cascade. |
| 6 | 33637963 | rs35564151 | 0.96 | 2 | 135372951 | G/A | 5.24E-08 | TMEM163 | Predicted to enable zinc ion binding activity; Predicted to be involved in zinc ion import into synaptic vesicle; Predicted to be located in early endosome membrane; Predicted to be active in intracellular vesicle and plasma membrane; Predicted to be integral component of synaptic vesicle membrane. |
| 7 | 31417202 | rs190982 | 0.94 | 5 | 88223420 | A/G | 2.8 × 10^−6^ | MEF2C | Has both trans-activating and DNA binding activities; Maintaining the differentiated state of muscle cells. |
| 8 | 31417202 | rs9473117 | 1.09 | 6 | 47431284 | A/C | 1.2 E-10 | CD2AP | Regulates the actin cytoskeleton; It is implicated in dynamic actin remodeling and membrane trafficking that occurs during receptor endocytosis and cytokinesis. |
| 9 | 31417202 | rs114812713 | 1.32 | 6 | 41034000 | G/C | 2.1 E-13 | OARD1 | A deacylase can convert O-acetyl-ADP-ribose to ADP-ribose and acetate, O-propionyl-ADP-ribose to ADP-ribose and propionate, and O-butyryl-ADP-ribose to ADP-ribose and butyrate. |
| 10 | 31417202 | rs75932628 | 2.08 | 6 | 41129252 | C/T | 2.7E-15 | TREM2 | Forms a receptor signaling complex with the TYRO protein tyrosine kinase binding protein; Functions in immune response and may be involved in chronic inflammation by triggering the production of constitutive inflammatory cytokines. |
| 11 | 31417202 | rs9331896 | 0.88 | 8 | 27467686 | T/C | 4.6E-24 | CLU | A secreted chaperone; Involved in several basic biological events such as cell death, tumor progression, and neurodegenerative disorders. |
| 12 | 31417202 | rs4735340 | 0.94 | 8 | 95976251 | T/A | 9.2E-08 | NDUFAF6 | It plays an important role in the assembly of complex I (NADH-ubiquinone oxidoreductase) of the mitochondrial respiratory chain through regulation of subunit ND1 biogenesis. |
| 13 | 31417202 | rs73223431 | 1.10 | 8 | 27219987 | C/T | 6.3E-14 | PTK2B | A cytoplasmic protein tyrosine kinase which is involved in calcium-induced regulation of ion channels and activation of the map kinase signaling pathway; It may represent an important signaling intermediate between neuropeptide-activated receptors or neurotransmitters that increase calcium flux and the downstream signals that regulate neuronal activity; it undergoes rapid tyrosine phosphorylation and activation in response to increases in the intracellular calcium concentration, nicotinic acetylcholine receptor activation, membrane depolarization, or protein kinase C activation; This protein has been shown to bind CRK-associated substrate, nephrocystin, GTPase regulator associated with FAK, and the SH2 domain of GRB2. |
| 14 | 33637963 | rs1878036 | 1.03 | 10 | 82280137 | T/G | 2.74E-09 | TSPAN14 | Enables enzyme binding activity; Involved in positive regulation of Notch signaling pathway, protein localization to plasma membrane and protein maturation; Located in plasma membrane. |
| 15 | 33637963 | rs1171814 | 0.98 | 10 | 61645833 | G/T | 3.80E-08 | CCDC6 | Function as a tumor suppressor. |
| 16 | 31417202 | rs3851179 | 0.88 | 11 | 85868640 | C/T | 6.0E-25 | PICALM | The protein is involved in AP2-dependent clathrin-mediated endocytosis at the neuromuscular junction. |
| 17 | 31417202; 33637963 | rs11218343 | 0.80 | 11 | 121435587 | T/C | 2.9E-12; 5.59E-14 | SORL1 | Plays roles in endocytosis and sorting. |
| 18 | 31417202; 33637963 | rs3740688 | 0.92 | 11 | 47380340 | T/G | 5.4E-13; 1.13E-10 | SPI1 | Activates gene expression during myeloid and B-lymphoid cell development; The nuclear protein binds to a purine-rich sequence known as the PU-box found near the promoters of target genes, and regulates their expression in coordination with other transcription factors and cofactors; The protein can also regulate alternative splicing of target genes. |
| 19 | 31417202; 33637963 | rs17125924 | 1.14 | 14 | 53391680 | A/G | 1.4E-09; 3.69E-10 | FERMT2 | Enables several functions, including actin binding activity; phosphatidylinositol-3,4,5-trisphosphate binding activity; and type I transforming growth factor beta receptor binding activity. Involved in several processes, including cell surface receptor signaling pathway; positive regulation of cell differentiation; and positive regulation of cellular component biogenesis. Acts upstream of or within cell adhesion and protein localization to cell junction. Located in cytosol; focal adhesion; and nucleoplasm. Is extrinsic component of cytoplasmic side of plasma membrane. Part of adherens junction and plasma membrane |
| 20 | 31417202 | rs12881735 | 0.92 | 14 | 92932828 | T/C | 7.4E-09 | SLC24A4 | It is a member of the potassium-dependent sodium/calcium exchanger protein family. |
| 21 | 31417202 | rs593742 | 0.93 | 15 | 59045774 | A/G | 6.8E-09 | ADAM10 | Members of the ADAM family are cell surface proteins with a unique structure possessing both potential adhesion and protease domains; Cleaves many proteins including TNF-alpha and E-cadherin. |
| 22 | 33637963 | rs117618017 | 1.09 | 15 | 63569902 | C/T | 1.05E-08 | APH1B | A multi-pass transmembrane protein that is a functional component of the gamma-secretase complex, which also contains presenilin and nicastrin. This protein represents a stabilizing cofactor for the presenilin holoprotein in the complex. The gamma-secretase complex catalyzes the cleavage of integral proteins such as notch receptors and beta-amyloid precursor protein. |
| 23 | 31417202 | rs10467994 | 0.94 | 15 | 51008687 | T/C | 4.3E-07 | SPPL2A | It is a member of the GXGD family of aspartic proteases, which are transmembrane proteins with two conserved catalytic motifs localized within the membrane-spanning regions, as well as a member of the signal peptide peptidase-like protease (SPPL) family. This protein is expressed in all major adult human tissues and localizes to late endosomal compartments and lysosomal membranes. |
| 24 | 31417202 | rs7185636 | 0.92 | 16 | 19808163 | T/C | 2.4E-08 | IQCK | It belongs to IQ motif-containing family of proteins. The IQ motif serves as a binding site for different EF-hand proteins such as calmodulin. |
| 25 | 31417202 | rs62039712 | 1.16 | 16 | 79355857 | G/A | 3.7E-08 | WWOX | It is a member of the short-chain dehydrogenases/reductases (SDR) protein family; induce apoptosis; Disruption of a similar gene in mouse results in impaired steroidogenesis, additionally suggesting a metabolic function for the protein |
| 26 | 33637963 | rs2884738 | 1.00 | 16 | 31126321 | C/A | 4.47E-09 | VKORC1 | It is responsible for the reduction of inactive vitamin K 2,3-epoxide to active vitamin K in the endoplasmic reticulum membrane. |
| 27 | 31417202; 33637963 | rs429358 | - | 19 | 45411941 | T/C | 1.2E-881; 0 | APOE | The protein is a major apoprotein of the chylomicron. It binds to a specific liver and peripheral cell receptor, and is essential for the normal catabolism of triglyceride-rich lipoprotein constituents. |
| 28 | 31417202 | rs6024870 | 0.88 | 20 | 54997568 | G/A | 3.5E-08 | CASS4 | Enables protein tyrosine kinase binding activity. Involved in several processes, including positive regulation of protein kinase B signaling; positive regulation of protein tyrosine kinase activity; and positive regulation of substrate adhesion-dependent cell spreading. Located in focal adhesion. Part of cytoplasm. |
| 29 | 31417202 | rs2830500 | 0.93 | 21 | 28156856 | C/A | 2.6E-08 | ADAMTS1 | It is a member of the ADAMTS (a disintegrin and metalloproteinase with thrombospondin motif) protein family; It may be associated with various inflammatory processes as well as development of cancer cachexia. |
| 30 | 33637963 | rs679515 | 0.95 | 1 | 207750568 | T/C | 1.40E-23 | CR1 | The protein mediates cellular binding to particles and immune complexes that have activated complement. Decreases in expression of this protein and/or mutations in this gene have been associated with gallbladder carcinomas, mesangiocapillary glomerulonephritis, systemic lupus erythematosus, sarcoidosis and Alzheimer's disease. |
| 31 | 31417202 | rs35868327 | 0.68 | 5 | 52665230 | T/A | 2.6E-07 | FST | Follistatin is a single-chain gonadal protein that specifically inhibits follicle-stimulating hormone release. |
| 32 | 31417202 | rs10808026 | 0.90 | 7 | 143099133 | C/A | 1.3E-10 | EPHA1 | EPH and EPH-related receptors have been implicated in mediating developmental events, particularly in the nervous system. |
| 33 | 31417202 | rs4723711 | 0.94 | 7 | 37844263 | A/T | 2.8E-07 | NME8 | The protein is implicated in ciliary function. |
| 34 | 31417202 | rs12539172 | 0.92 | 7 | 100091795 | C/T | 9.3E-10 | NYAP1 | Predicted to be involved in neuron projection morphogenesis and phosphatidylinositol 3-kinase signaling. |
| 35 | 33637963 | rs1859788 | 0.91 | 7 | 99971834 | A/G | 3.28E-18 | PILRA | Cell signaling pathways rely on a dynamic interaction between activating and inhibiting processes. SHP-1-mediated dephosphorylation of protein tyrosine residues is central to the regulation of several cell signaling pathways. Two types of inhibitory receptor superfamily members are immunoreceptor tyrosine-based inhibitory motif (ITIM)-bearing receptors and their non-ITIM-bearing, activating counterparts. Control of cell signaling via SHP-1 is thought to occur through a balance between PILRalpha-mediated inhibition and PILRbeta-mediated activation. These paired immunoglobulin-like receptor genes are located in a tandem head-to-tail orientation on chromosome 7. The ITIM-bearing member of the receptor pair, which functions in the inhibitory role. |
| 36 | 31417202 | rs792072 | 0.94 | 10 | 11720308 | A/G | 1.8E-11 | ECHDC3 | Predicted to enable enoyl-CoA hydratase activity. Involved in positive regulation of cellular response to insulin stimulus. Predicted to be active in mitochondrion. |
| 37 | 31417202 | rs7933202 | 0.89 | 11 | 59936926 | A/C | 1.9E-19 | MS4A2 | The beta subunit of the high affinity IgE receptor which is a member of the membrane-spanning 4A gene family. Members of this nascent protein family are characterized by common structural features and similar intron/exon splice boundaries and display unique expression patterns among hematopoietic cells and nonlymphoid tissues. |
| 38 | 31417202 | rs7295246 | 1.06 | 12 | 43967677 | T/G | 3.9E-07 | ADAMTS20 | The protein is a member of the ADAMTS family of zinc-dependent proteases. The protein has a signal peptide that is cleaved to release the mature peptide, which is secreted and found in the extracellular matrix. This protein may be involved in tissue remodeling. |
| 39 | 33637963 | rs12444183 | 0.95 | 16 | 81773209 | A/G | 5.46E-08 | PLCG2 | The protein is a transmembrane signaling enzyme that catalyzes the conversion of 1-phosphatidyl-1D-myo-inositol 4,5-bisphosphate to 1D-myo-inositol 1,4,5-trisphosphate (IP3) and diacylglycerol (DAG) using calcium as a cofactor. IP3 and DAG are second messenger molecules important for transmitting signals from growth factor receptors and immune system receptors across the cell membrane. |
| 40 | 31417202 | rs138190086 | 1.32 | 17 | 61538148 | G/A | 7.5E-09 | ACE | It is an enzyme involved in blood pressure regulation and electrolyte balance. It catalyzes the conversion of angiotensin I into a physiologically active peptide angiotensin II. Angiotensin II is a potent vasopressor and aldosterone-stimulating peptide that controls blood pressure and fluid-electrolyte balance. This angiotensin converting enzyme (ACE) also inactivates the vasodilator protein, bradykinin. Accordingly, the encoded enzyme increases blood pressure and is a drug target of ACE inhibitors, which are often prescribed to reduce blood pressure. This enzyme additionally plays a role in fertility through its ability to cleave and release GPI-anchored membrane proteins in spermatozoa. |
| 41 | 33637963 | rs2526378 | 1.02 | 17 | 56404349 | A/G | 3.07E-07 | TSPOAP1 | Enables benzodiazepine receptor binding activity. Predicted to be involved in regulation of presynaptic cytosolic calcium ion concentration. Located in mitochondrion. |
| 42 | 33637963 | rs12151021 | 1.08 | 19 | 1050874 | A/G | 2.41E-13 | ABCA7 | The protein is a member of the superfamily of ATP-binding cassette (ABC) transporters. The function of this protein is not yet known; however, the expression pattern suggests a role in lipid homeostasis in cells of the immune system. |
| 43 | 33637963 | rs3865444 | 0.97 | 19 | 51727962 | C/A | 1.29E-08 | CD33 | Enables protein phosphatase binding activity and sialic acid binding activity. Involved in several processes, including negative regulation of cytokine production; negative regulation of monocyte activation; and positive regulation of protein tyrosine phosphatase activity. Located in several cellular components, including Golgi apparatus; external side of plasma membrane; and peroxisome. |

Table S2 Epitope of target proteins encoded by candidate genes

| **Epitope No.** | **Gene Abbreviations** | **Protein names** | **Epitope** |
| --- | --- | --- | --- |
| 1 | ADAM10 | ADAM metallopeptidase domain 10 | CFSDEFKVETSNKVLDYDTSHIYTGH |
| 2 | ADAMTS1 | ADAM metallopeptidase with thrombospondin type 1 motif 1 | SLKCLSHDGGVLSHESGDPLKKPK |
| 3 | BIN1 | bridging integrator 1 | VKESDWNQHKKCRGVFPENFTERVP |
| 4 | CASS4 | Cas scaffold protein family member 4 | CSDELAFSRGDILTILEQHVPESEGR |
| 5 | CD2AP | CD2-associated protein | YIVEYDYDAVHDDELTIRVGEIIRNVKKC |
| 6 | CLU | Clusterin | TQGEDQYYLRVTTVASHTSDSDVPSGC |
| 7 | FERMT2 | FERM domain containing kindlin 2 | CGIRMPDGCYADGTWELSVHVTDLNR |
| 8 | INPP5D | Inositol polyphosphate-5-phosphatase | CPPLPVKSPAVLHLQHSKGRDYRDNTEL |
| 9 | IQCK | IQ motif containing K | PSHIVRLKPSCSTDSSFTRTPVPTV |
| 10 | MEF2C | Myocyte enhancer factor 2C | SPIGLTRPSPDERESPSVKRMRLSEC |
| 11 | NDUFAF6 | NADH: ubiquinone oxidoreductase complex assembly factor 6 | ASAHGSVWGPLRLGIPGLCDRRPPLGL |
| 12 | OARD1 | O-acyl-ADP-Ribose deacylase 1 | SSLNEDPEGSRITYVKGDLFACPKTRSL |
| 13 | PICALM | Phosphatidylinositol-binding clathrin assembly protein | CQPTLIYSQPVMRPPNPFGPVSG |
| 14 | PTK2B | Protein tyrosine kinase 2β | CSGVSEPLSRVKLGTLRRPEGPAEPM |
| 15 | SLC24A4 | Solute carrier family 24 member 4 | CASGLFGSLGHKTASASKRVLPDTW |
| 16 | SORL1 | Sortilin related receptor 1 | CEVWTQRLHGGSAPLPQDRGFLVVQGDPR |
| 17 | SPPL2A | Signal peptide peptidase like 2A | EAILHASGNGTTKDYCMLYNPYWTH |
| 18 | TREM2 | Triggering receptor expressed on myeloid cells 2 | CPLRLLILLFVTELSGAHNTTVFQG |
| 19 | WWOX | WW domain containing oxidoreductase | CKDTDSEDELPPGWEERTTKDG |
| 20 | ADAMTS4 | ADAM metallopeptidase with thrombospondin type 1 motif 4 | LHDNSKPCISLNGPLSTSRHVMAPVMA |
| 21 | APH1B | Aph-1 homolog B, gamma-secretase subunit | CRSLKLSLLSQDKNFLLYNQRSR |
| 22 | APOE | Apolipoprotein E | KLEEQAQQIRLQAEAFQARLKSWFEPLVC |
| 23 | CCDC6 | Coiled-coil domain containing 6 | QPTVPSAATSQPTPSQHSAHPSSQC |
| 24 | NCK2 | NCK adaptor protein 2 | VEHYKKAPIFTSEHGEKLYLVRALC |
| 25 | SPRED2 | Sprouty related EVH1 domain containing 2 | MTEETHPDDDSYIVRVKAVVMTRRC |
| 26 | TMEM163 | Transmembrane protein 163 | CIVVKAIHDLSTRLLPEVDDFLF |
| 27 | TSPAN14 | Tetraspanin 14 | GVPFSSSVPDPAQKVVNTQCGYDVRIQ |
| 28 | VKORC1 | Vitamin K epoxide reductase complex subunit 1 | KAARARDRDYRALSDVGTAISCSRV |

Table S3: Analysis of the inter-assay deviation between plates

| **Epitope No.** | **Target proteins** | **Plate #1** | **Plate #2** | **Plate #3** | **Plate #4** | **Plate #5** | **Plate #6** | **Plate #7** | **Mean** | **SD** | **CV(%)** |
| --- | --- | --- | --- | --- | --- | --- | --- | --- | --- | --- | --- |
| 1 | ADAM10 | 0.340 | 0.436 | 0.426 | 0.409 | 0.415 | 0.489 | 0.479 | 0.428 | 0.046 | 10.723% |
| 2 | ADAMTS1 | 0.427 | 0.488 | 0.483 | 0.539 | 0.355 | 0.445 | 0.466 | 0.458 | 0.053 | 11.655% |
| 3 | BIN1 | 0.401 | 0.490 | 0.548 | 0.510 | 0.403 | 0.533 | 0.502 | 0.484 | 0.055 | 11.319% |
| 4 | CASS4 | 0.383 | 0.450 | 0.547 | 0.462 | 0.374 | 0.499 | 0.444 | 0.451 | 0.056 | 12.492% |
| 5 | CD2AP | 0.351 | 0.409 | 0.420 | 0.376 | 0.433 | 0.532 | 0.437 | 0.423 | 0.053 | 12.581% |
| 6 | CLU | 0.391 | 0.347 | 0.439 | 0.351 | 0.456 | 0.453 | 0.379 | 0.402 | 0.043 | 10.796% |
| 7 | FERMT2 | 0.470 | 0.414 | 0.453 | 0.407 | 0.448 | 0.474 | 0.379 | 0.435 | 0.033 | 7.590% |
| 8 | INPP5D | 0.392 | 0.522 | 0.411 | 0.447 | 0.368 | 0.534 | 0.433 | 0.444 | 0.058 | 13.163% |
| 9 | IQCK | 0.423 | 0.499 | 0.409 | 0.469 | 0.350 | 0.536 | 0.444 | 0.447 | 0.057 | 12.703% |
| 10 | MEF2C | 0.396 | 0.497 | 0.496 | 0.534 | 0.396 | 0.545 | 0.555 | 0.488 | 0.062 | 12.708% |
| 11 | NDUFAF6 | 0.446 | 0.468 | 0.463 | 0.520 | 0.394 | 0.513 | 0.553 | 0.480 | 0.049 | 10.240% |
| 12 | OARD1 | 0.446 | 0.449 | 0.469 | 0.417 | 0.433 | 0.503 | 0.407 | 0.446 | 0.030 | 6.717% |
| 13 | PICALM | 0.375 | 0.410 | 0.447 | 0.373 | 0.409 | 0.459 | 0.373 | 0.407 | 0.033 | 8.116% |
| 14 | PTK2B | 0.428 | 0.414 | 0.434 | 0.386 | 0.443 | 0.467 | 0.380 | 0.422 | 0.029 | 6.855% |
| 15 | SLC24A4 | 0.461 | 0.455 | 0.469 | 0.410 | 0.472 | 0.494 | 0.452 | 0.459 | 0.024 | 5.152% |
| 16 | SORL1 | 0.517 | 0.423 | 0.543 | 0.507 | 0.568 | 0.517 | 0.620 | 0.528 | 0.056 | 10.654% |
| 17 | SPPL2A | 0.516 | 0.444 | 0.620 | 0.533 | 0.519 | 0.508 | 0.510 | 0.521 | 0.048 | 9.199% |
| 18 | TREM2 | 0.531 | 0.454 | 0.545 | 0.599 | 0.558 | 0.603 | 0.663 | 0.565 | 0.061 | 10.844% |
| 19 | WWOX | 0.504 | 0.411 | 0.570 | 0.490 | 0.597 | 0.579 | 0.597 | 0.536 | 0.065 | 12.050% |
| 20 | ADAMTS4 | 0.388 | 0.385 | 0.382 | 0.481 | 0.459 | 0.462 | 0.410 | 0.424 | 0.039 | 9.230% |
| 21 | APH1B | 0.426 | 0.409 | 0.384 | 0.521 | 0.425 | 0.472 | 0.407 | 0.435 | 0.043 | 9.895% |
| 22 | APOE | 0.436 | 0.447 | 0.411 | 0.452 | 0.425 | 0.453 | 0.379 | 0.429 | 0.025 | 5.744% |
| 23 | CCDC6 | 0.428 | 0.464 | 0.403 | 0.419 | 0.440 | 0.453 | 0.403 | 0.430 | 0.022 | 5.093% |
| 24 | NCK2 | 0.442 | 0.407 | 0.401 | 0.485 | 0.446 | 0.487 | 0.462 | 0.447 | 0.032 | 7.076% |
| 25 | SPRED2 | 0.404 | 0.386 | 0.504 | 0.509 | 0.591 | 0.556 | 0.525 | 0.496 | 0.070 | 14.130% |
| 26 | TMEM163 | 0.468 | 0.410 | 0.585 | 0.541 | 0.538 | 0.506 | 0.608 | 0.522 | 0.063 | 12.061% |
| 27 | TSPAN14 | 0.456 | 0.426 | 0.571 | 0.562 | 0.508 | 0.477 | 0.593 | 0.513 | 0.059 | 11.470% |
| 28 | VKORC1 | 0.458 | 0.382 | 0.572 | 0.505 | 0.584 | 0.549 | 0.595 | 0.521 | 0.072 | 13.845% |
